# The Role of Non-Structural Protein NSs in the Pathogenesis of Severe Fever with Thrombocytopenia Syndrome

**DOI:** 10.3390/v13050876

**Published:** 2021-05-11

**Authors:** Jumana Khalil, Hiroki Kato, Takashi Fujita

**Affiliations:** 1 Laboratory of Virus Immunology, Institute for Frontier Life and Medical Science, Kyoto University, Kyoto 606-8507, Japan; jumana.atkhalil.86c@st.kyoto-u.ac.jp (J.K.); hkato@uni-bonn.de (H.K.); 2 Laboratory of Molecular and Cellular Immunology, Graduate School of Biostudies, Kyoto University, Kyoto 606-8507, Japan; 3 Institute of Cardiovascular Immunology, University Hospital Bonn, University of Bonn, 53127 Bonn, Germany

**Keywords:** SFTSV NSs, NF-κB, cytokine storm, TBK1, viral pathogenesis, interferons, innate immunity

## Abstract

Viral non-structural proteins, such as NSs of the newly emerging severe fever with thrombocytopenia syndrome virus, are well established virulence factors, mediating viral pathogenesis and disease progression through various mechanisms. NSs has been described as a potent interferon antagonist and NF-κB agonist, two divergent signaling pathways in many immune responses upon a viral encounter. In this review, we highlight the many mechanisms used by NSs on the host that promote viral replication and hyper-inflammation. Understanding these host-pathogen interactions is crucial for antiviral therapy development.

## 1. Introduction

Severe fever with thrombocytopenia syndrome virus (SFTSV), also recently called Dabie virus (DABV), is a newly discovered *banyangvirus* in the family *Phenuiviridae* [1]. It causes severe viral hemorrhagic fever with a mortality rate as high as 30% [2,3]. SFTSV infection is often characterized by acute fever, myalgia, and gastrointestinal symptoms. These symptoms are accompanied by thrombocytopenia and leukocytopenia, which cause bleeding and have severe consequences. In response to this heavy disease burden, in addition to the lack of efficient vaccine or treatment, the World Health Organization lists SFTSV as one of the top prioritized pathogens for research and development for effective treatment [4]. The high fatality rate can be attributed to virus-induced cytokine storms, coagulopathies caused by disseminated intravascular coagulation, and the progression of multi-organ dysfunction to multi-organ failure [5,6].

The non-structural protein NSs, which is encoded by the small segment of the SFTSV ambisense genome, is the main virulence factor for the virus. NSs was shown to control innate immune responses of the host and to facilitate viral replication. These observations suggest that NSs plays an important role in the disease progression.

Although our understanding of the host–virus interactions and pathogenesis of SFTSV is limited, many studies have investigated these mechanisms and the development of antiviral measures. In this review, we provide a collective picture of the role of NSs in SFTSV infection.

## 2. Influence of NSs on Innate Immunity

### 2.1. Induction of Interferon

Interferons (IFNs) are secreted cytokines that play various roles in innate and adaptive immune responses and classified into three types: I, II, and III [7]. IFNs represent the first line of defense against invading viral pathogens. Type I IFNs are characterized by their strong antiviral activities, which limit viral replication and spread. Type II IFNs also share antiviral activities, but these are more prominent in immune responses against bacteria and parasites [7,8]. The receptors of type I and II IFNs are ubiquitously expressed; however, the expression of type III IFN receptors is tissue-specific. Normally the expression of type I and III IFN genes are suppressed, but they are induced by viral infection.

Intracellular and endosomal pattern recognition receptors, such as RIG-I-like receptors (RLRs) and toll-like receptors (TLRs), recognize viral RNA and trigger downstream signaling that ultimately results in the expression of IFNs and inflammatory cytokines [9]. In the case of SFTSV, RIG-I was shown to be the main receptor detecting the viral genome in the cytoplasm of infected cells. TLR3 also participates in SFTSV recognition, but MDA-5 contributes only minimally to this function [10,11]. Downstream of RIG-I and TLR-3, MAVS and TRIF, respectively, mediate the induction of IFN through TBK1 and IκB kinase (IKK) activation [12]. The anti-IFN function of NSs was first described in 2012 [13]. However, the mechanism behind IFN antagonism is still being investigated, although SFTSV NSs may disrupt the IFN pathway at multiple steps [11,12,13,14,15,16]. SFTSV NSs has the unique property of forming cytoplasmic granules, in which NSs entraps several host proteins and factors, blocking their functions. The E3 ubiquitin ligase TRIM25 is entrapped by NSs, hindering the TRMI25-mediated ubiquitination of RIG-I, a step required for its activation, and inhibiting the antiviral IFN production at the early level of PRR [11]. Additionally, the critical protein kinase TBK1 is physically trapped into the NSs granule, thus activation of IRF-3 is blocked [14]. Whether SFTSV also directs its antagonism against IRF7-mediated IFN-α production was also addressed. IRF-7 interacts directly with NSs in transfected and SFTSV-infected cells [12]. Collectively, NSs is a strong inhibitor of IFN production.

Notably, NSs interaction with TBK1 was also reported in mice [15], but not yet in other species. In contrast, STAT2 antagonism by NSs is absent in mice [15]. It will be merited if future research addresses species-specific functions of NSs protein.

### 2.2. Action of Interferon

**Figure 1:**
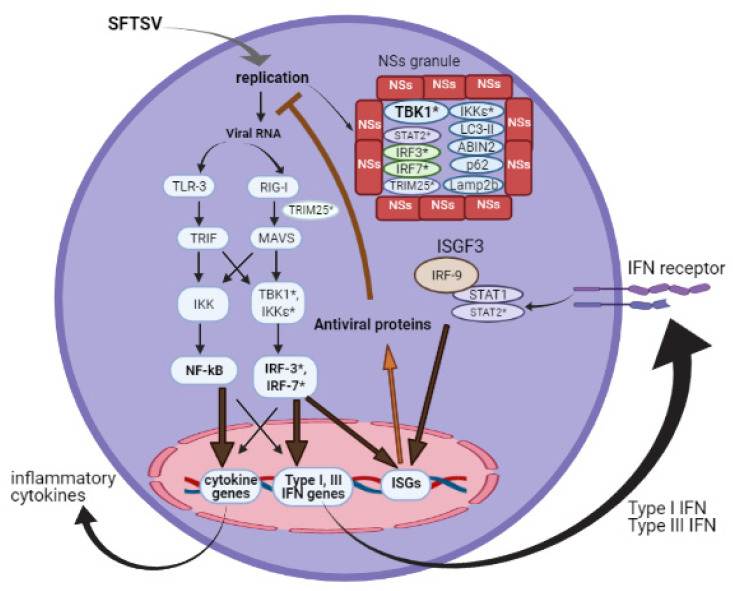
Type I IFN antagonism by SFTSV NSs. In virus-infected cells, various viral RNA species are sensed by TLR3 and RLR to initiate a signaling cascade for antiviral responses. NSs protein can intrinsically form cytoplasmic granules that entrap several factors involved in antiviral responses (* indicates such factors).

Type I and III IFNs share a common signaling pathway, culminating in the activation of IFN-stimulated gene factor 3 (ISGF3), a heterotrimer consisting of the signal transducer and activator of transcription 1 (STAT1) and STAT2 heterodimer and IFN regulatory factor 9 (IRF-9). ISGF3 transcriptionally regulates numerous IFN-stimulated genes (ISGs) that encode a wide range of antiviral proteins to establish an antiviral state. Type II IFN signaling, however, involves STAT1 homodimers as the downstream transcription complex. Thus, while STAT1 protein is required for the induction of all types of IFNs, STAT2 is not involved in type II IFN-induced signaling. NSs directly interacts with STAT2 and sequesters it into NSs granules [16]. The interaction of STAT1 with NSs is controversial [17,18,19], as is the role of type II IFN in anti-SFTSV function [18,20,21]. Figure 1 summarizes the viral induction of type I and III IFN production and action, and the NSs antagonism.

### 2.3. Non-IFN Antiviral Responses

Autophagy has divergent roles in viral infections; depending on the virus, it may restrict or enhance viral replication [22].

Preliminary results concerning SFTSV and autophagy have been reported. The autophagy-related protein LC3-II negatively regulates SFTSV replication [23]. NSs interacts with LC3-II and co-localizes with the autophagy marker p62 and the autolysosome marker Lamp2b [23,24]. p62 is a substrate for TBK1 [25], which is implicated in autophagy regulation. It is also reported that NSs enhances p62 stability and oligomerization by inhibiting tripartite motif 21 (TRIM21) [24]. However, the role of autophagy in SFTSV infection has not been clarified.

### 2.4. Role of NSs in Viral Replication

It has been hypothesized that SFTSV NSs is directly involved in viral RNA replication by acting as a scaffold protein, forming a platform, which may be critical in host-virus interactions, as well as viral replication. The group named this platform viroplasm-like structures (VLS), referring to the microscopic cytoplasmic granules formed by SFTSV NSs [26], and the replication complex consists of a lipid droplet-based structure [26,27]. Cell cycle arrest through a NSs/cyclin-dependent kinase 1 (CDK1) interaction may enhance SFTSV replication [28].

## 3. SFTSV Infection Mouse Models

SFTSV infection is not fatal in wild-type (WT) mice, but it is in mice deficient of type I IFN receptor (*Ifnar1*−/−) [29]. This effect is consistent with type I IFN being a strong barrier against the virus. However, this assumption ignores the fact that SFTSV encodes NSs, a potent antagonist against type I IFN responses. Therefore, the mechanism of the fatal SFTSV infection in *Ifnar1*−/− mice is not simple. Indeed, the expression of inflammatory cytokines and chemokines is markedly enhanced in *Ifnar1*−/− compared with WT mice [10]. Notably, *Mavs*−/− and *Myd88*−/− mice, which exhibit impaired type I IFN and inflammatory cytokine production, show comparable survival to WT mice against SFTSV infection. These results strongly suggest that hyper-inflammation, or a cytokine storm, is responsible for the fatal outcome of the infection, although a mechanistic link between the lack of IFNAR1 and the hyper-inflammation remains to be investigated.

## 4. Cytokine Storm Induction

The induction of a cytokine storm by SFTSV was described shortly after the virus was first identified. SFTS patients, especially fatal cases, exhibit abnormally high cytokine levels in their sera, which correlates with multi-organ damage and poor prognosis. Elevated cytokines include pro-inflammatory mediators such as tumor necrosis factor-α (TNF-α), interleukin-6 (IL-6), C-X-C motif ligand 10 (CXCL10), and monocyte chemoattractant protein [20,21,30,31,32,33]. These results are consistent with SFTSV infection in *Ifnar1−/−* mice [10]. The hyper-inflammation seen in the mouse model was characterized by an enhanced production of proinflammatory cytokines and chemokines at the mRNA level, including CXCL1, CCL2, CCL3, TNF-α, IL-1β and IL-6 genes.

**Figure 2:**
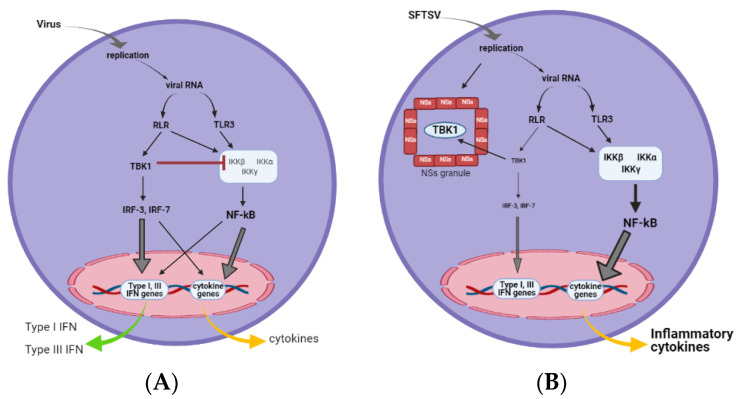
NSs induction of an NF-κB-mediated cytokine storm. (**A**) In virus-infected cells, viral RNA sensing activates TBK1, which activates IRF-3 for the transcriptional activation of type I IFN and cytokine genes. TBK1 negatively regulates the IKK complex by interacting with IKKβ to limit the inflammatory responses mediated by NF-κB. (**B**) Upon SFTSV infection, viral NSs granules sequester TBK1, resulting in impaired IFN production. This liberates IKK from the negative regulation of TBK1, leading to the hyper-activation of NF-κB and inflammatory responses.

NSs expression in vitro, in conjugation with double-stranded RNA or the virus-induced signal, augmented the induction of pro- and anti-inflammatory cytokines, a response reminiscent of a cytokine storm [32,33], suggesting that SFTSV induces the cytokine storm through NSs [32]. It has been recently shown that NF-κB may be the primary transcription factor mediating the NSs-induced cytokine upregulation and that controlling NF-κB activity may be a therapeutic approach to treat SFTS [32]. The study revealed that TBK1 has intrinsic inhibitory activity for IKKβ, a component of the IKK complex. Genetic deletion or the pharmacological inhibition of TBK1 resulted in the hyper-activation of NF-κB. Because NSs is a strong inhibitor of TBK1, its expression resulted in the release of NF-κB from TBK1 suppression. The function of NSs in IFN response suppression and hyper-inflammation is summarized in Figure 2. Studies in vivo and in vitro have highlighted TBK1 as a critical regulator of the anti-viral and inflammatory responses upon SFTSV infection. These results suggest the suppression of NF-κB as a promising therapy for SFTS [10,32].

Intriguingly, immune-suppressive cytokines are also upregulated throughout the course of SFTSV infection, and their upregulation is associated with disease severity. These anti-inflammatory cytokines include IL-10, IL-12, and IL-23 [34]. One report found that NSs interacts with ABIN2 (A20-binding inhibitor of NF-κB activation-2) to stabilize the TPL2 (tumor progression locus 2)-ABIN2-p105 ternary complex and activate the TPL2 signaling pathway, which promotes robust IL-10 expression [33]. The TPL2 signaling pathway is required for the SFTSV-induced lethal phenotype [33]. These findings, however, question whether the outcome of SFSV infection is immunopathological or immunosuppressive. Determining which outcome dominates the pathological scenario in human SFTS requires more study.

Another report linked the SFTSV-induced IL-10 upregulation to the expression of miR-146b, an endogenous non-coding RNA that modulates the expression of specific genes post-transcriptionally. MiR-146b targets STAT1 to skew macrophage differentiation into anti-inflammatory M2 type during the late stages of the infection [35]. SFTSV replicates higher in the presence of M2 than in M1 [35]; thus, this skewed differentiation of macrophages may have an important role in the pathogenesis of SFTSV.

## 5. Conclusions

In this review, we addressed recent progress in host responses against SFTSV and the role of the main virulence factor for this virus, NSs, in the viral pathogenesis. We explain how NSs aggravates the disease progression by interacting with host proteins inside NSs-formed cytoplasmic granules. NSs interacts with TBK1, leading to the suppression of anti-viral IFN production and a hyper-activation that causes pro-inflammatory cytokine storms. This effect gives the virus a state of uncontrolled replication inside its target hematopoietic cells. NSs also interacts with several other proteins (summarized in Table 1) inside the granules to manipulate immune signaling pathways and evade anti-viral responses. This characteristic of SFTSV NSs is unique and has not been reported among other *Phenuiviridae*. Comparably, NSs protein of Rift Valley fever virus (RVFV NSs) assembles into nuclear and cytosolic aggregates in infected cells. In the nucleus, it forms filamentous structure. While in the cytosol, amorphous granules are observed [36,37].

Notably, other effects of SFTSV on host cell machinery have been reported. However, the role of NSs in such interference is not yet identified. For example, SFTSV triggers mitochondrial dysfunction and NLRP3 inflammasome activation [38]. Moreover, the virus activates p38 mitogen-activated protein kinase (MAPK) signaling pathway [39], which plays a crucial role in many biological processes, such as cell proliferation and apoptosis, inflammation, aging and tumorigenesis [40,41,42]. Additionally, SFTSV causes a global disruption of naïve T-cell differentiation, and antibody class switching [34], resulting in impeded humoral immune responses. Whether NSs is the viral component regulating such effects is worth investigating in the future.

In conclusion, NSs is the virulent factor for SFTSV that mediates numerous pathological processes in the host. Based on its role in the pathogenesis of SFTSV, we envision a therapy directed specifically against NSs to alleviate the syndrome.

## Figures and Tables

**Table 1:** List of host proteins to which NSs interacts.

Protein	Effect of Interaction
TBK1	Suppressed IFN responses and activated NF-κB signaling [14,32]
LC3-II	Promoted viral replication and autophagy regulation during SFTSV infection [23]
TRIM25	Inhibited ubiquitination and activation of RIG-I [11]
IRF3	Inhibited transcription of IFN-I [14]
IRF7	Inhibited transcription of IFN-I [12]
STAT2	Suppressed JAK/STAT signaling and abrogated ISG production [16,17]
IKKε	Suppressed IFN responses [14]
CDK1	Cell cycle arrest at G2/M transition [28]
ABIN2	Activated TPL2 signaling and IL-10 production [33]
p62	Unknown role in autophagy regulation [24]
Lamp2b	Unknown role in autophagy regulation [23]

## Data Availability

Not applicable.
